# The Games Radicals Play: Special Issue on Free Radicals and Radical Ions

**DOI:** 10.3390/molecules20022831

**Published:** 2015-02-09

**Authors:** John C. Walton, Ffrancon Williams

**Affiliations:** 1 EaStCHEM School of Chemistry, University of St. Andrews, St. Andrews KY16 9ST, UK; 2 Department of Chemistry, University of Tennessee, Knoxville, TN 37996-1600, USA

Chemistry and Physics have aptly been described as “most excellent children of Intellect and Art” [1]. Both these “children” engage with many playthings, and molecules rank as one of their first favorites, especially radicals, which are amongst the most lively and exciting. Checking out radicals dancing to the music of entropy round their potential energy ballrooms is surely both entertaining and enlightening. Radicals’ old favorite convolutions are noteworthy, but the new styles, modes and arrangements appearing on the scene are even more interesting. Some of these are ephemeral and enjoy only a brief appearance, others are retro-types reappearing in new guises, still others are genuinely new and “go viral” in the scientific world. This Special Issue of *Molecules* contains the observations and reflections of a select group of chemists and physicists fascinated by this spectacle. It contains an eclectic mix reflecting on new modes and advances as well as on permutations and combinations that revive mature themes. 

Many types of radicals rapidly couple with suitably accoutered acceptor partners. On encountering an alkene the outcome may well be a recurrent cycle in which more and more alkene is assimilated into a growing polymer chain. Resolving what was going on in these processes played a major part in the acceptance of free radicals as key players in organic chemistry. Furthermore the polymer products have found many valuable uses in textiles, as structural materials and in electronic devices. It is no surprise, therefore, to find the polymerization theme well represented in this Special Issue. Instead of polymer, the outcome of a radical and acceptor encounter may be a conveniently functionalized product. When the radical is so constructed that the acceptor group forms part of its own architecture; their encounter results in ring closure; with formation of cycles or heterocycles. These can be attractive functionalization methods because they require no corrosive acids or bases and often do not need wasteful protection/deprotection protocols. Several developments of unusual radical and radical-ion addition and cyclization processes are included in the Special Issue. 

A useful and selective method of generating radicals involves irradiating designer precursors with light of appropriate wavelength. One of the most exciting recent discoveries is that low intensity visible light can be employed for this purpose provided certain photocatalysts are included. Apart from the safety of avoiding hard UV radiation, the possibility of thereby harnessing more of the sun’s energy is obvious. This approach is proving hugely popular and the range of effective chemical systems and suitable photocatalysts is rapidly expanding [2,3]. Research into the development of applicable homogeneous photocatalysts as well as of heterogeneous semiconductor photocatalysts also features in this issue. 

One technique that has particularly empowered this area of science is EPR spectroscopy. Because of their paramagnetism, the structures and dynamic behavior of radicals and radical ions can be studied in amazing detail be means of this technique. How EPR spectroscopy can reveal the most intricate details of the structural permutations of small molecules, particularly when working hand in hand with computationally executed quantum mechanical theory, figures in several articles in the Special Issue. At the other extreme of the molecular scale, pulsed dipolar EPR methods are also rapidly evolving for structural measurements on large proteins and polynucleotides. For study in this way the biomolecules need to be labeled with one, two, or more stable radicals or paramagnetic transition metals. Practically a whole new branch of science is emerging as radicals suitable for this purpose, as well as methods of making them and attaching them to biomolecules, are being discovered. Not only does this Special Issue contain key aspects of this science, but an overview of preparations of special yardstick biradicals and polyradicals of known structure for calibration purposes is also presented. 

Reactive Oxygen Species (ROS), and the Oxidative Stress they cause, is on most of the lists of “things to worry about.” It is reassuring therefore to read in the Special Issue that one of our favorite beverages, coffee, contains an array of antioxidant components seemingly designed to help mitigate this disorder. 

The resurgence in radical chemistry and physics witnessed over the last few decades owes as much to radical ions as it does to neutral radicals. The former are generated with great ease by single electron transfers (SET) to stable organic molecules in gaseous, liquid and solid phases. That radical ions play important roles in biological processes has long been known. EPR studies of aromatic radical anions and stable semidione radical anions played key roles in the development of semi-empirical and ab initio quantum mechanical methods applicable to organic chemical structures. Recently, intriguing new roles with potential technological importance have been discovered for radical ions as polarons and bipolarons in conducting polymers. The importance of radical ions in various aspects of organo-catalysis is drawing increased attention. Remarkably, organic electron donors—metal-free organic reagents—capable of generating radicals and anions [4] are now being deployed in organic synthesis; particularly the Super-SET organic donors developed by Murphy and co-workers [5].

In this context, the recently discovered electron-transfer mediated radical coupling of iodoarenes to styrene in the presence of the *t*-butoxide anion represents a most interesting novel reaction with potential utility in the medicinal field. The exact nature of the initiation step in this chain reaction has remained elusive, however, but the article by Murphy and colleagues in this Special Issue sheds new light on this process.

It was originally postulated that the well-established step of dissociative electron transfer to the iodoarene leading to the propagating aryl radical occurred directly from the *t*-butoxide anion. Murphy’s contribution shows that this cannot be the mechanism since virtually no reaction takes place in the complete absence of nitrogen-containing triggering agents such as various phenanthrolines. Most significantly, Murphy reports that a mixture of the *t*-butoxide anion with dialkyldiketopiperazines functions as a super electron donor, the most likely active agent being the diamagnetic enolate anion generated by proton transfer from the diketopiperazine with the *t*-butoxide anion acting as a strong base. 

These studies nicely exemplify the fascinating interplay that can exist between diamagnetic species such as enolate anions on the one hand, and paramagnetic intermediates involving both neutral aryl radicals and arene radical anions on the other hand. This novel chemistry induced by the *t*-butoxide anion clearly widens the scope of many potentially useful radical and radical ion reactions.

A paper by Rhydderch and Howe supplements the liquid-phase EPR studies showing that radical ions and radicals are strongly implicated in the reactions of organic compounds induced by semiconductor photocatalysis with titania [3,6]. It breaks new ground with EPR studies at 80 and 4 K aimed at probing the primary processes that occur directly at the solid surface. Most of the species detected under these cryogenic conditions give rise to anisotropic EPR signals that can be largely assigned to the initial products of electron or hole transfer at the solid-liquid interface. They conclude that the species observed in solution under homogeneous steady-state conditions result from the desorption of the initially-formed adsorbed species from the surface of the photocatalyst.

The course of True Research never did run smooth [7] so it is not surprising to find some obstacles to be overcome and some unfinished business amongst the reports. However, optimism is the oxygen of innovation and the ozone of discovery and plenty of this is also on display. We are grateful to all the contributors for the interesting new molecular structures and transformations they sent in. It is our hope that you will find some enjoyment and that some intriguing ideas will be engendered as you read in this Special Issue of the entropic games radicals play. 
